# Introgression of pigs in Taihu Lake region possibly contributed to the improvement of fertility in Danish Large White pigs

**DOI:** 10.1186/s12864-023-09860-x

**Published:** 2023-12-04

**Authors:** Chenxi Liu, Ruihua Huang, Guosheng Su, Liming Hou, Wuduo Zhou, Qian Liu, Zijian Qiu, Qingbo Zhao, Pinghua Li

**Affiliations:** 1 Institute of Swine Science (Key Laboratory of Pig Genetic Resources Evaluation and Utilization, Ministry of Agriculture and Rural Affairs (Nanjing)), Nanjing Agricultural University, Nanjing, 210095 China; 2 Huaian Academy, Nanjing Agricultural University, Huaian, 223001 China; 3 Center for Quantitative Genetics and Genomics, Aarhus University, Aarhus, DK-8000 Denmark

**Keywords:** Taihu Lake region pigs, Danish Large White pigs, Introgression, Fertility, *NDUFS4* gene

## Abstract

**Background:**

Eurasian pigs have undergone lineage admixture throughout history. It has been confirmed that the genes of indigenous pig breeds in China have been introduced into Western commercial pigs, providing genetic materials for breeding Western pigs. Pigs in Taihu Lake region (TL), such as the Meishan pig and Erhualian pig, serve as typical representatives of indigenous pig breeds in China due to their high reproductive performances. These pigs have also been imported into European countries in 1970 and 1980 s. They have played a positive role in improving the reproductive performances in European commercial pigs such as French Large White pigs (FLW). However, it is currently unclear if the lineage of TL pigs have been introgressed into the Danish Large White pigs (DLW), which are also known for their high reproductive performances in European pigs. To systematically identify genomic regions in which TL pigs have introgressed into DLW pigs and their physiological functions, we collected the re-sequencing data from 304 Eurasian pigs, to identify shared haplotypes between DLW and TL pigs.

**Results:**

The findings revealed the presence of introgressed genomic regions from TL pigs in the genome of DLW pigs indeed. The genes annotated within these regions were found to be mainly enriched in neurodevelopmental pathways. Furthermore, we found that the 115 kb region located in SSC16 exhibited highly shared haplotypes between TL and DLW pigs. The major haplotype of TL pigs in this region could significantly improve reproductive performances in various pig populations. Around this genomic region, *NDUFS4* gene was highly expressed and showed differential expression in multiple reproductive tissues between extremely high and low farrowing Erhualian pigs. This suggested that *NDUFS4* gene could be an important candidate causal gene responsible for affecting the reproductive performances of DLW pigs.

**Conclusions:**

Our study has furthered our knowledge of the pattern of introgression from TL into DLW pigs and the potential effects on the fertility of DLW pigs.

**Supplementary Information:**

The online version contains supplementary material available at 10.1186/s12864-023-09860-x.

## Background

Wild boars are primarily categorized into Asian and European variations. Through both natural and artificial selection, they have evolved into the present-day Asian and European domestic pigs [1]. Regarding Asian domestic pigs, there are a diverse range of Chinese indigenous pig breeds, each with distinct characteristics. These breeds can be further classified into three groups: Taihu Lake region indigenous pigs (TL), Southern China indigenous pigs (SCD) and Southwestern China indigenous pigs (SWCD) [2]. TL pigs exhibit distinct characteristics compared to other Asian pig breeds, particularly in terms of their remarkable fertility. They display outstanding farrowing capabilities, excellent mothering instincts, and a notably high count of teats [3, 4]. Although the geographical distribution of Eurasian pigs is widely dispersed, there is lineage admixture between them as a result of human trade or political activities. This includes the trade between China and Europe, which has led to the introduction of indigenous pigs from Southern and Eastern China into the UK and other region. There are also pig breeds such as Meishan and Jiaxing Black that have been introduced to France, Japan and other countries since the 1970s [5]. Therefore, it is highly likely that the current Western commercial pigs may contain some genomic regions that are introgessed from TL pigs and perform important functions.

Since 1992, the improvement of farrowing performance has become one of the main objectives in Danish pig breeding. This selection had led to an average increase of 0.30 piglets/litter per year for DLW pigs, resulting in an average litter size of 15.3 piglets born alive in 2007 [6]. Furthermore, over the last decade, along with the widespread use of genomic selection techniques and an increased emphasis on the breeding significance of farrowing performance has contributed to a more substantial improvement in the farrowing performance of DLW pigs [7, 8]. This rapid advancement in selection can be attributed not only to the great improvement in breeding accuracy, but also may to the introgression of high-farrowing genotypes or haplotypes for selection. Additionally, it has been found that genomic regions from the TL pigs have introgressed into populations such as French Large White and Netherlandish Large White pigs to improve their farrowing performance. Furthermore, the *AHR* gene [9] and *KATNAL1* gene [10] have been identified as important introgressed genes for the improvement of the reproductive capabilities of both sows and boars. Research conducted on Danish pigs has revealed that the Danish Duroc breeding population did indeed show introgression of genomic regions from Meishan pigs, and genes such as *NR6A1* had been identified as important introgressed genes [11]. This study indicated that there could be a significant likelihood that Danish pig breeds were indeed mixed with TL lineage.

Although all European Large White pigs originated from the UK, due to long-term artificial selection in different directions, the genetic backgrounds of different strains may differ significantly between each other. Although researchers have identified important introgressed signs from TL pigs in French and Netherlandish Large White pigs, it is not clear if the DLW pigs are also introgressed, and investigating the introgressed genomic regions and related biological functions of DLW pigs would be worthwhile.

## Results

### DLW pigs specific genetic structure

**Fig. 1:**
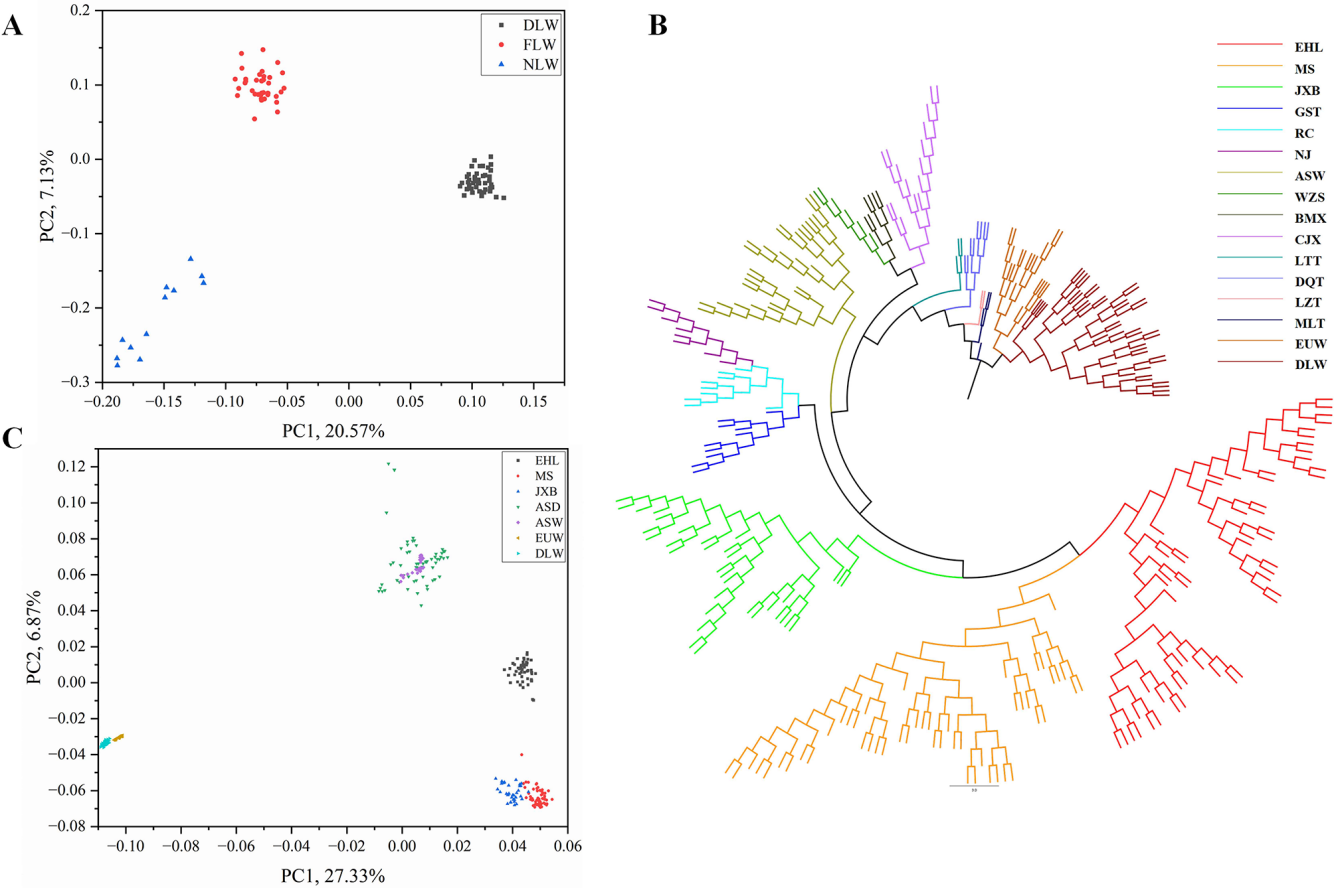
Population relationship and structure. (**A**) Principal component analysis of Danish Large White pigs (DLW), French Large White pigs (FLW) and Netherlandish Large White pigs (NLW). (**B**) Neighbor-joining tree among individuals. The following breeds are in clockwise order: EHL, Erhualian pig; MS, Meishan pig; JXB, Jiaxing Black pig; GST, Gansu Tibetan pig; RC, Rongchang pig; NJ, Neijiang pig; ASW, Asian wild boar; WZS, Wuzhishan pig; BMX, Bamaxiang pig; CJX, Congjiangxiang pig; LTT, Litang Tibetan pig; DQT, Diqing Tibetan pig; LZT, Linzhi Tibetan pig; MLT, Milin Tibetan pig; EUW, European wild boar; DLW, Danish Large White pigs. (**C**) Principal component analysis of Chinese and European pigs. ASD, Asian domestic pig

To investigate the specific genetic structure of DLW pigs, we conducted a principal component analysis (PCA) comparing FLW pigs, NLW pigs and DLW pigs (Fig. 1A). The PCA result revealed that DLW pigs clustered separately from FLW and NLW pigs, indicating significant genetic differences among these three strains of LW pigs. This divergence in genetic structure may explain the unique production characteristics of DLW pigs.

To elaborate on the variations in genetic structure between Chinese and Western pig breeds, specifically between DLW and Taihu Lake region (TL) pigs, we combined 304 re-sequencing data from domestic and wild pigs in Eurasia. Subsequently, we conducted an Neighbor-joining phylogenetic (NJ) tree analysis (Fig. 1B). The analysis revealed the distinct clustering of Chinese and Western pig breeds. Asian wild boars and domestic pigs clustered together, while European wild boars and Danish Large White pigs formed a separate cluster. Within the Asian pig clusters, indigenous pigs in Taihu Lake region exhibited a more independent clustering pattern, which is consistent with previous studies [2]. The Eurasia pigs PCA diagram revealed similar trends (Fig. 1C), where European wild boar (EUW) and DLW pigs clustered on the left side of the diagram, while Asian domestic and wild boar clustered in the upper right corner, and TL pigs clustered in the lower left corner. The results indicated that DLW pigs exhibited a closer genetic structure to EUW pigs, aligning with the breeding history of DLW pigs. DLW pigs demonstrated greater dissimilarity from Asian domestic pigs and wild boar, particularly when compared to TL pigs.

### Introgression pattern of TL pigs into DLW pigs

**Fig. 2:**
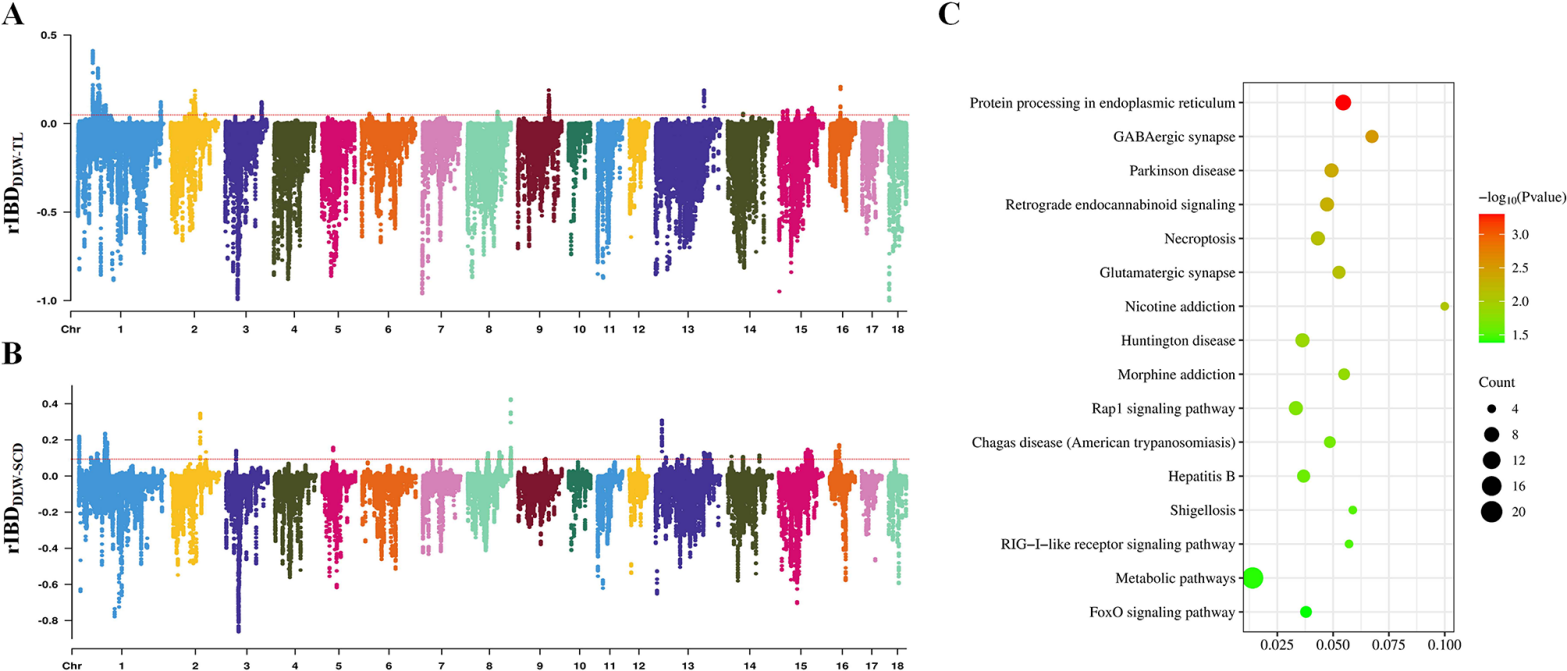
Introgressed haplotypes in Danish Large White (DLW) pigs. (**A**) Manhattan plot of relative identity-by-descent (rIBD) values between DLW and Taihu lake region (TL) pigs (positive value) or EUW (negative value). The red dashed line indicates the top 0.5% significance threshold. (**B**) Manhattan plot of rIBD values between DLW and Southern Chinese (SCD) pigs (positive value) or EUW (negative value). The red dashed line indicates the top 0.5% significance threshold. (**C**) Significantly enriched KEGG pathways of introgressed genes in the introgressed regions from TL into DLW pigs

In order to determine if there was an introgression of lineage between high-farrowing DLW pigs and TL pigs, we conducted an relative identity-by-descent (rIBD) analysis between DLW pigs, EUW pigs and TL pigs. The results showed that the genome of DLW pigs consistently shared more haplotypes (rIBD < 0) with EUW pigs. The average rIBD value for all windows was − 0.073 (Fig. 2A, Supplementary Table S1). However, we also discovered large genomic regions on SSC1, SSC2, SSC3, SSC9, SSC13 and SSC16, which displayed a greater consistency in shared haplotypes between DLW and TL pigs, with rIBD peak values surpassing 0.128. In fact, some peaks even exceeded 0.4 (Supplementary Table S1). The data indicated that the genome of DLW pigs, particularly in the relevant regions mentioned above, was likely introgressed with the lineage of TL pigs. We identified regions with the highest 0.5% rIBD values as potential regions of introgression. Ultimately, we identified 37 introgressed regions (Supplementary Table S1), which comprised 0.48% of DLW pigs genome.

We annotated the introgressed regions and identified a total of 283 protein-coding genes. Initially, we compared the introgressed genes between DLW pigs and FLW pigs or NLW pigs, which have been confirmed to be introgressed with TL pigs’ lineage [9, 10] (Supplementary Figure S1). It was observed that the genes introgressed from TL into DLW pigs were mostly different from those introgressed from TL into FLW or NLW pigs. The majority of the introgressed genes (256 out of 283) were newly discovered. Among these, some genes, such as *ME1* associated with meat quality and *PGRMC2* associated with fertility, were found to be located in introgressed regions in multiple Large White populations.

To further determine whether the introgressed genomic regions or protein-coding genes in DLW pigs were introgressed specifically from TL pigs, rather than introgressed from widespread Asian pigs, particularly domestic pigs in Southern China, we performed rIBD analysis between Southern China indigenous pigs (SCD), EUW pigs, and DLW pigs (Fig. 2B, Supplementary Table S2). Additionally, the first 0.5% window of rIBD values was considered as potential introgressed regions. We discovered that the overall average rIBD value for SCD pigs (average rIBD = -0.029) was higher compared to that of TL pigs (average rIBD = -0.073). It was shown that DLW pigs exhibited a higher degree of introgression with the SCD pigs’ lineage, which was consistent with the breeding history of Large White pigs. The introduction of Southern China indigenous pigs to Europe on a large scale initiated around 1680s, contributing genetic resources to facilitate the selection and breeding of Large White pigs in the UK, the ancestors of the present-day Large White pigs in different countries [12]. The patterns of introgression from TL pigs into DLW pigs differed from those of SCD pigs into DLW pigs (Supplementary Figure S2). Specifically, 238 out of 283 genes were annotated within the introgressed region originating from TL pigs only. This inconsistent introgression regions or genes may serve different functions. In fact, SCD pigs exhibit a high resistance to disease [5] and the relevant introgressed regions were more likely to be associated with selection for disease resistance in pigs, whereas the introgressed regions from TL pigs were more likely to be associated with traits such as reproduction [3–5].

To initially identify the biological functions of these introgressed genes, we annotated them for functional enrichment using the KEGG database. The majority of pathways that introgressed genes enriched in were associated with neural and brain development (Fig. 2C, Supplementary Table S3). For example, these introgressed genes were enriched in several synapse-related pathways, such as GABAergic synapse [13], Parkinson disease [14]. TL pigs are very maternal and docile [15], and the related introgressed regions or genes may affect the neurodevelopment of DLW pigs. Focusing on the most significant peaks (rIBD peak > 0.128) located in SSC1, SSC2, SSC9, SSC13, SSC16, many of the genes have been reported to have functional effects on neurodevelopment (Supplementary Table S4). For example, the *GRIK2* gene may underlie diverse neurodevelopmental disorders [16]. Additionally, genes related to reproductive performances have also been reported. The *KLHL32* gene is reported to be related to duck reproduction and nervous system [17].

In addition, for the results of significantly enriched KEGG pathway, it has been reported that Retrograde endocannabinoid signaling pathway could reduce GABAergic synaptic transmission to gonadotropin-releasing hormone neurons, GnRH [18]. GnRH plays important regulatory roles in reproductive behaviour. Furthermore, FoxO signaling pathway has been reported to be significant pathways contributing to high farrowing rates in Meishan pigs [19]. This indicated that this pathways may have impact on fertility.

### The introgressed *NDUFS4* haplotype from TL pigs increasing fertility of DLW pigs

**Fig. 3:**
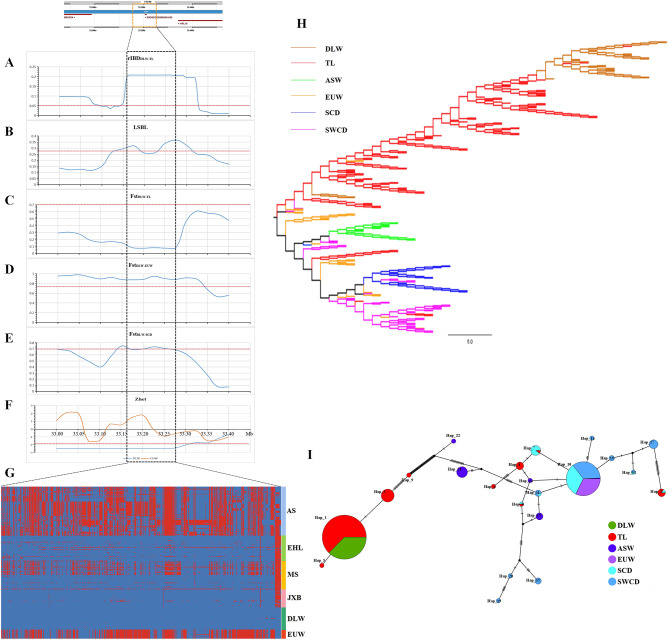
Introgression pattern in SSC16: 33,160 kb − 33,275 kb region. (**A**) The relative identity-by-descent (rIBD) values between DLW and TL and EUW pigs. The red dashed line indicates the top 0.5% threshold. (**B**) Locus-specific branch length (LSBL) analysis between TL pigs and other Asian domestic or wild pigs. The red dashed line indicates the significant threshold (Z test, P < 0.05). (**C**)-(**E**) Genetic differentiation coefficients (Fst) analysis between DLW pigs and Taihu Lake region (TL) pigs, European wild boar (EUW) pigs and Southern China indigenous pigs (SCD) pigs respectively. The red dashed line indicates the significant threshold (Z test, P < 0.05). (**F**) Z-transformed heterozygosity (Zhet) statistics between DLW and EUW pigs. The red dashed line indicates the significant threshold (Z test, P < 0.05). (**G**) Haplotype heat map in this 115 kb region. Major and minor alleles in DLW pigs are indicated by blue and red, respectively. AS, Asian domestic and wild pigs. (**H**) Neighbor-joining tree among individuals for this 115 kb region. SWCD, Southwestern China indigenous pigs. (**I**) Haplotype network map in this 115 kb region. Each circle represents a haplotype, and the size of the circle is proportional to the haplotype frequency. The line length represent the difference between haplotypes. Different colors represent pigs from different geographical regions

Focusing on rIBD peaks, we discovered one significant peak located in SSC16 (mean rIBD = 0.2055, 33,160 kb − 33,275 kb) was more likely to have introgressed from TL into DLW pigs and had an impact on the farrowing performance of DLW pigs. Initially, we analyzed the rIBD values for this particular region, and found that the frequency of shared IBD haplotypes between DLW and TL pigs in this 115 kb region was significantly higher compared to surrounding regions (Fig. 3A). Furthermore, we observed a notable increase in genetic differentiation (locus-specific branch length, LSBL) between TL pigs and other Asian domestic pigs as well as wild boars (Fig. 3B). This suggested that TL pigs possessed distinct haplotypes in the 115 kb genomic regions. Additionally, we noticed an increased genetic differentiation coefficients (Fst) values between DLW and EUW pigs in this region (Fig. 3D), as well as a similar trend between DLW and SCD pigs (Fig. 3E). Conversely, an decreased Fst values between DLW and TL pigs was visibly observed (Fig. 3C). These findings clearly demonstrate that DLW and TL pigs in this region share a distinct haplotype.

If the haplotype of this 115 kb region was indeed introgressed from TL pigs into DLW pigs and had functions on fertility, then this region would be subjected to strong artificial selection. As a consequence, the causal loci were selected for favorable allele frequencies. Additionally, due to the “hitchhiking effect” caused by linkage disequilibrium, this genomic region could evolve in DLW pigs towards favorable haplotypes. Consequently, lower levels of polymorphism would be observed. Based on this, we calculated Z-transformed heterozygosity (Zhet) values for DLW pigs and EUW pigs in this region respectively to measure the heterozygosity of this region in the two populations. It was discovered that DLW pigs exhibited less polymorphism compared to EUW pigs (Z test, *P* < 0.01) (Fig. 3F). This suggests that this region in the DLW pigs experienced higher selection pressure.

In order to further clarify the differences between the 115 kb genomic region in diverse pig breeds, we extracted SNPs from this region in Eurasian pig breeds, including TL, DLW and other pig breeds, and phased them to construct a haplotype heat map (Fig. 3G). Our findings indicated that Asian domestic pigs, wild boars, and EUW pigs displayed highly consistent haplotypes, whereas TL and DLW pigs exhibited opposite haplotypes. Moreover, we observed that individuals from Jiaxing Black (n = 34) and Erhualian pigs (n = 50) maintained almost all haplotypes consistent with LW pigs. However, within the Meishan pigs, some individuals (22 out of 56) showed inconsistent haplotypes. This suggests that the introgressed regions in DLW pigs were more likely to have originated from Jiaxing Black or Erhualian pigs. We also conducted NJ tree analysis on this region (Fig. 3H), and discovered that DLW and TL pigs formed a single large cluster, while the remaining Eurasian pigs formed another large cluster, with the exception of few Meishan individuals. This suggests that DLW pigs and TL pigs have a closer genetic distance in this region. Finally, a haplotype network map was constructed for this region (Fig. 3I). To identify representative SNPs and decrease the number of haplotype, we screened 104 informative SNPs. This allowed us to extract representative haplotypes. It was discovered that the majority haplotypes of DLW pigs (N_haplotype_ = 74), clustered together with the major haplotype of TL pigs (N_haplotype_ = 124) and formed hap_1. The remaining Eurasian pigs, including SCD (N_haplotype_ = 38), Southwestern China indigenous pigs (SWCD) (N_haplotype_ = 41), EUW (N_haplotype_ = 38) clustered on the opposite side and formed the haplotype hap_10, which differed significantly from hap_1. The hap_10 was surrounded by numerous small frequency haplotypes, including hap_16, hap_18, and so on (Fig. 3I, Supplementary Table S5). These results once again suggest that within this 115 kb region, DLW and TL pigs share a unique genetic structure pattern that is different from any other Eurasian pigs.

**Fig. 4:**
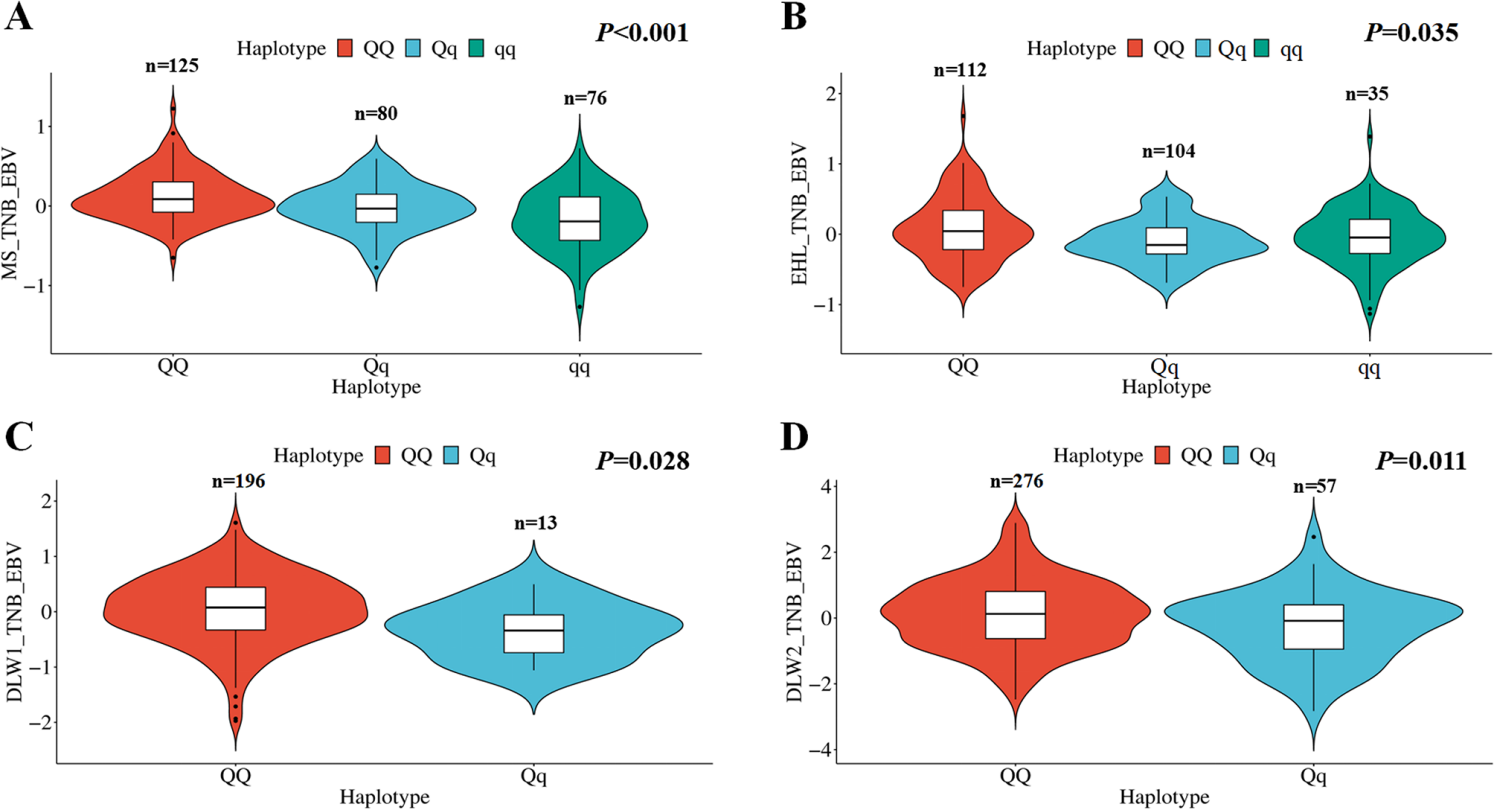
Haplotype association analysis of candidate introgressed region located in SSC16: 33,160 kb − 33,275 kb. (**A**)-(**D**) Association analysis between estimated breeding values for total litter size trait (TNB_EBV) of sows and homozygous (QQ) or others (Qq and qq) for the introgressed haplotypes. The one-way ANOVA was used to compute the *P*-value. (**A**) was the result for Meishan pig (MS, *P* < 0.001), (**B**) was the result for Erhualian pig (EHL, *P* = 0.041), (**C**) was the result for population 1 of Danish Large White pig (DLW1, *P* = 0.028) and (**D**) was the result for population 2 of Danish Large White pig (DLW2, *P* = 0.011) respectively. N is the sample size of corresponding haplotypes

To investigate whether the 115 kb region haplotypes located in SSC16 were associated with farrowing performance in TL and FLW pigs, we collected phenotypic data on total number of litter size (TNB) from Meishan pigs (n = 281), Erhualian pigs (n = 251), and Danish Large White pigs DLW-1 (n = 209) and DLW-2 (n = 333) from two Chinese breeding farms. We calculated EBV of total number of born (TNB_EBV) separately for each farm. We then constructed haplotypes and performed association analysis between TNB_EBV and the haplotypes. The results showed that individuals with homozygote of the introgressed haplotype (QQ), where Q is the major haplotype of TL pigs, had significantly higher TNB_EBV compared to individuals with heterozygotes (Qq) and homozygote of the unintrogressed haplotype (qq) in both Meishan (*P* < 0.001) (Fig. 4A, Supplementary Table S6) and Erhualian pigs (*P* = 0.035) (Fig. 4B, Supplementary Table S6). For DLW pigs in two different farms, individuals with the introgressed haplotype (QQ), showed significantly higher TNB_EBV compared to individuals with heterozygote genotype (Qq) (*P* = 0.028 for DLW-1; *P* = 0.011 for DLW-2) (Fig. 4C and D, Supplementary Table S6). We observed that there were no individuals with the qq haplotype in DLW pigs, and that the QQ haplotype was prevalent, indicating continuous selection for favorable alleles and gradual purification of the major allele in DLW pigs. This finding is consistent with the low heterozygosity observed in DLW pigs in this region, suggesting strong selection pressure.

Three protein-coding genes, namely *NDUFS4*, *ARL15*, and *ENSSSCG00000041656*, were annotated targeting the 115 kb introgressed region of SSC16. Among these genes, the *ENSSSCG00000041656* gene is located exactly within the region. This gene encodes an ovule protein, which is an important reproduction-related protein. The begg database (https://bgee.org/) [20] revealed that this gene was most highly expressed in the testis, as well as in reproductive organs or cells such as granulosa cells and oocytes (FDR < 0.05). Moreover, the *NDUFS4* gene has been reported to be associated with several reproductive processes, including embryonic development [21]. In contrast, there are no reports associating the *ARL15* gene with reproduction. This suggests that *ENSSSCG00000041656* and *NDUFS4* were more likely to influence farrowing performance in DLW pigs based on their reported gene function.

**Fig. 5:**
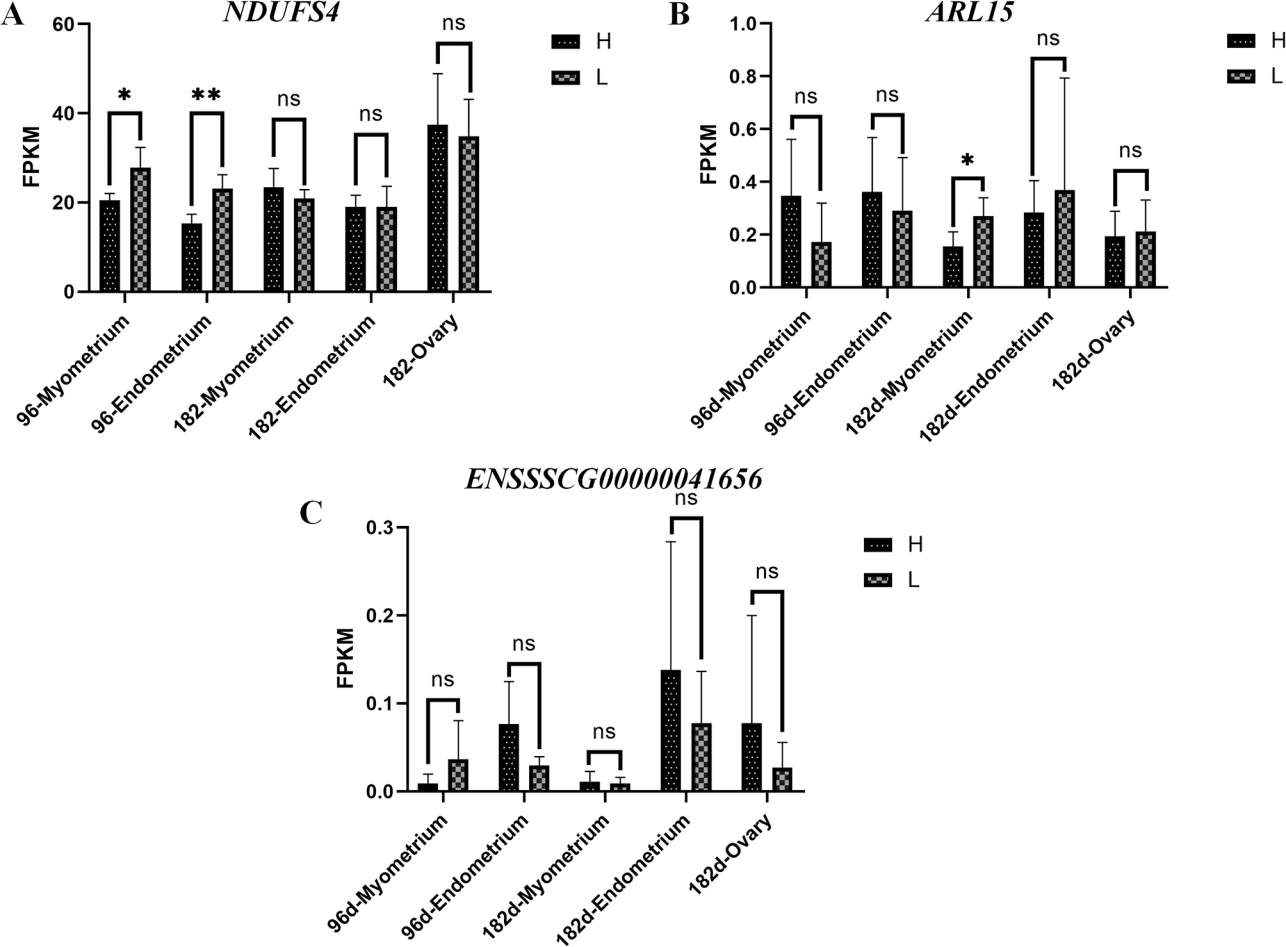
Expression patterns of candidate genes located around SSC16: 33,160 kb − 33,275 kb region. The results of gene expression differences between high and low farrowing Erhualian pigs’ uterus and ovarian tissue for multiple time points were (**A**), (**B**), and (**C**) which corresponded to *NDUFS4*, *ARL15*, and *ENSSSCG00000041656* gene, respectively. H represents high farrowing Erhualian group and L represents low farrowing Erhualian group. * represents a significant difference (*P* < 0.05) and ** represents a significant difference (*P* < 0.01). NS represents difference not significant

To further validate the functions of the three protein-coding genes located around this 115 kb introgressed region, we collected samples of endometrial and myometrium tissue from extreme high and low farrowing Erhualian pigs at 96 days, as well as samples of endometrium, myometrium, and ovaries at 182 days. Next, we utilized RNA-seq to identify differences in expression patterns of the *NDUFS4*, *ARL15*, and *ENSSSCG00000041656* genes between the extreme high and low farrowing Erhualian pigs’ reproduction-associated tissues. The expression of *NDUFS4* gene was found to be significantly higher in the myometrium of the extreme low-farrowing group at 96 days compared to the high-farrowing group (*P* = 0.023) (Fig. 5A). Similarly, for the 96 day endometrial tissue, the *NDUFS4* gene was expressed significantly higher in the extreme low-farrowing group compared to the high-farrowing group (*P* = 0.006) (Fig. 5A). This suggests that the *NDUFS4* gene plays an important role in the development of the uterus in Erhualian pigs at 96 days. Additionally, among the three genes mentioned, the *NDUFS4* gene exhibited the highest expression levels, with high expression observed in both uterine and ovarian tissues at different stages (FPKM value ranging from 13.95 to 45.35) (Fig. 5A). Combined with the reported functions in embryonic development, the *NDUFS4* gene was considered an important candidate gene for fertility. The *ARL15* gene showed significantly higher expression in the 182 day myometrium of the extreme low-farrowing group compared to the high-farrowing group (*P* = 0.039) (Fig. 5B). *ENSSSCG00000041656* did not exhibit any differential expression pattern in the reproductive tissues at any stage and had extremely low expression levels, with FPKM values below 0.2 (Fig. 5C). Together, we observed a specific region introgressed from TL pigs into DLW pigs and identified important candidate genes, such as the *NDUFS4* gene, that play a role in fertility.

## Discussion

All European Large White pigs originated in the UK. However, due to the different selection and breeding directions, diversity strains of Large White pigs have been formed during the centuries-long breeding time. Significant stratification exists between these strains, including French Large White (FLW) pigs, Netherlandish Large White (NLW) pigs and Danish Large White (DLW) pigs. Previous research confirmed genomic regions from the TL pigs have introgressed into populations such as French Large White and Netherlandish Large White pigs to improve their farrowing performance [9, 10]. In this study, we also have identified haplotypes that have introgressed from TL into DLW pigs. It is important to note that introgression from Chinese indigenous pigs into Western pigs has occurred. During the Qing Dynasty, the Chinese government engaged in normal commercial trade with European countries. As a result, indigenous pigs from Southern and Eastern China were introduced into the UK to support the breeding of British Large White pigs. In the 1970 and 1980 s, the Chinese government officially introduced Meishan pigs, Jiaxing Black pigs and Jinhua pigs to several countries, including France, Hungary and the UK [5]. This prompted extensive research and a series of crossbreeding trials between TL pigs and Western commercial pigs, particularly in France [5]. The findings revealed that the progeny of these crosses exhibited reproductive performances that were comparable to TL pigs, suggesting that there was a hybrid advantage in reproductive performances when TL pigs were crossed with European commercial pig breeds.

Since 1992, the improvement of farrowing performance has become one of the main objectives in Danish pig breeding, the farrowing performance of DLW pigs have been improved greatly [6]. Besides the improvement of breeding accuracy for farrowing performance [7, 8], it is also possible that DLW pigs have been introgressed with high-farrowing genotypes or haplotypes, and through long-term directed selection and retention, the frequency of advantageous genotypes or haplotypes gradually accumulates and stabilizes in DLW pigs, ultimately impacting their farrowing performance. Also, TL pigs have demonstrated the highest farrowing performance among Chinese indigenous pig breeds. Considering the significant contribution of TL pigs to the genetic improvement of commercial pigs in European [9, 10], we investigated if the high farrowing performance of DLW pigs could be partly attributed to the introduction of lineage from TL pigs. As a result, the rIBD-based analysis confirmed that TL pigs’ lineage have introgressed into DLW pigs. Furthermore, the annotated protein-coding genes within introgressed region in DLW pigs were found to be primarily enriched in KEGG functions related to neurodevelopmental pathways, such as the GABAergic synapse and Parkinson’s disease [13, 14]. Neurodevelopment-related pathways related genes underwent a process of selection and may be associated with maternal behavior in the sow. Additionally, TL pigs possess high fertility, which is not only reflected in its high farrowing performance, but also in its strong maternal nature, docile character and strong ability to nurse its young. Studies have shown that TL pigs demonstrates positive maternal qualities, resulting in heavier piglets at the time of weaning and higher survival rate among piglets compared to Western pigs [22]. These genes may have introgressed from TL pigs to DLW pigs, enhancing maternal qualities in DLW pigs. Besides, we have also identified significantly enriched KEGG pathways, such as the FoxO signaling pathway, have been reported to be associated with fertility in pigs, particularly Meishan pigs [19]. This further enhances our understanding of the physiological function of the rIBD regions shared between TL and DLW pigs.

We also found that the genes introgressed into DLW pigs were mostly inconsistent with the genes introgressed into FLW pigs or NLW pigs. In fact, Meishan pigs have mainly been introduced to France and possibly supporting the genetic resources for the selection of French pig breeds [5]. It has been observed that FLW pigs exhibit more consistent haplotypes with Meishan pigs in the introgressed regions [10]. However, our findings indicate that the introgressed regions of DLW pigs, which display more consistent haplotypes with Erhualian and Jiaxing Black pigs. Although Meishan pig, Erhualian pig and Jiaxing Black all belong to TL pigs, there is a clear population stratification within them [2], and the genes that control reproductive performance in these pig breeds may not be identical. Moreover, the rIBD analyses of NLW pigs covered not only Meishan pigs, but also other populations like Jianquhai and Xiang breeds. These breeds exhibit relatively low reproductive performance and are not included in the TL pigs [5]. Additionally, there is a significant population stratification between Meishan pigs and Jianquhai or Xiang breeds. Consequently, the introgressed regions identified in NLW pigs may vary from those we identified using only representative TL pigs. In addition, the breeding goals of the three populations mentioned above are not the same. For example, DLW pigs were primarily focused on selecting for litter size traits. On the other hand, FLW pigs were selected for litter size traits as well as other traits such as teat number. These distinct breeding goals led to genomes that were subject to selection in different regions and different directions. Consequently, this might have resulted in changes in the frequency of haplotypes in the introgressed regions.

Focusing on the identified introgressed regions, we found a significant peak located in SSC16 and annotated *NDUFS4* as an important candidate gene for reproductive performance. There were evidences that: (1) the *NDUFS4* gene was located in the rIBD peak region (mean rIBD = 0.2055, 33,160 kb − 33,275 kb). (2) This region can distinguish TL pigs from other Asian pigs and may explain TL-specific traits such as high fecundity. (3) In the Fst analysis of diverse pig populations, it was discovered that DLW and TL in this region had lower Fst values, in contrast to higher Fst values between DLW pigs and other Eurasian pigs. (4) This region exhibited lower polymorphism in DLW pigs (average Zhet = -2.45), whereas EUW had higher polymorphism in this region (average Zhet = 0.36), suggesting that DLW pigs may be undergoing strong selection. (5) NJ tree, haplotype heatmap and haplotype clustering map in this region revealed that DLW pigs had closer clustering relationships or more consistent haplotypes with TL pigs, compared to other pig breeds. (6) Haplotype association analysis in this region revealed that the introgressed Q haplotype, which is the major haplotype in TL pigs, can improve TNB_EBV in various populations such as Meishan pigs, Erhualian pigs, and DLW pigs. (7) A total of three protein-coding genes, *NDUFS4*, *ARL15*, and *ENSSSCG00000041656*, were annotated around this region. Only the *NDUFS4* gene was highly expressed in reproduction-related tissues such as the uterus and ovary, and differentially expressed in multiple reproductive tissues between extreme high and low farrowing Erhualian pigs. The above results suggest that *NDUFS4* may be an important candidate gene affecting the reproductive performance of DLW pigs, whose advantageous haplotype was introgressed from TL pigs. In fact, *NDUFS4* encodes a nuclear-encoded accessory subunit of the mitochondrial membrane respiratory chain NADH dehydrogenase. This gene has been reported to be associated with embryonic development and hormone synthesis [21].

By integrating representative re-sequencing data from TL pigs, DLW pigs, and other Eurasian pig breeds, we have identified several rIBD regions that show high consistency among TL pigs and DLW pigs. One particular region, located in SSC16: 33,160 kb − 33,275 kb, is especially notable. Additionally, we have confirmed previous findings of introgressed genomic regions from TL pigs into Western pig breeds, such as Netherlandish Large White, French Large White, and Danish Duroc pigs [9–11]. This provides further evidence of lineage admixture between European and Chinese indigenous pig breeds, partly highlighting the genetic contribution of Chinese indigenous pig breeds to Western pigs. Notably, we have also discovered, for the first time, genomic evidence of TL pig haplotypes being introgressed into DLW pigs. However, further molecular biology analyses are required to validate and elucidate the possible biological roles of relevant candidate regions and genes in the development of high fertility traits in DLW pigs.

## Conclusions

Our study examined the introgressed pattern from TL into DLW pigs based on rIBD analysis, with the implementation of re-sequencing data-set. We observed that 0.48% of DLW pigs’ genome have been introgressed from TL pigs. The introgressed genes potentially impact the neural system development and fertility of DLW pigs. Through multi-omics analyses, including comparative genomics, quantitative genetics, and transcriptomics, we have identified the *NDUFS4* gene was an important candidate introgressed gene. The introgressed *NDUFS4* gene was strongly selected among DLW pigs, and could have a contribution to improved farrowing performance in various pig populations. These findings facilitate understanding the rapid and substantial increase in the farrow performance of DLW pigs.

## Methods

### Animal population

This study collected re-sequencing data from 304 Eurasian pigs. Among the 304 pigs, a total of 106 TL pigs, which included 56 Meishan pigs and 50 Erhualian pigs, as well as 44 Danish Large White pigs (DLW), were sequenced in this study (Supplementary Table S7), and the re-sequencing data for the remaining 154 Eurasian pigs were obtained from the pig Farm animal GTEx (FarmGTEx) project [23]. These includes the followings: Jiaxing Black pig in TL (34 individuals), Southern China indigenous pigs (SCD) consisting of Bamaxiang pig (6 individuals), Congjiangxiang pig (16 individuals), Neijiang pig (9 individuals), and Wuzhishan pig (6 individuals), Southwestern China indigenous pigs (SWCD) consisting of Tibetan pig (27 individuals) and Rongchang pig (8 individuals). Additionally, re-sequencing data for European wild boar (EUW; 19 individuals) and Asian wild boar (ASW; 29 individuals) are also included. Details of pig breeds information are shown in Supplementary Table S8.

### Whole-genome sequencing and SNP calling

For the 150 individuals sequenced in this study, DNA were extracted by the chloroform/phenol method. Then Illumina paired-end DNA libraries with an insert size of 350 bp were constructed using a Genomic DNA Sample Prep Kit (Illumina, USA) following the manufacturer’s protocols. The libraries were then sequenced on the Illumina NovaSeq PE150 platform (USA) in paired-end mode (2 × 150 bp). The average genome sequencing coverage of each individual was 11.2X, with the average genome coverage 98.03% (Supplementary Table S7). We used FASTA file of Sus_scrofa_11.1 as reference genome sequence. For sequencing data quality control, FastQC software (FastQC, RRID:SCR_014583) [24] was implemented with default parameters. We applied BWA [25] to blast reads against the reference genome and obtained binary BAM files from SAM files by means of SAMtools v1.4 (SAMTOOLS, RRID:SCR_002105) [26]. The steps of duplicate marking, base quality recalibration and duplicated reads removal were conducted by Picard v1.119 (PICARD, RRID:SCR_002105), GATK v4.0 (GATK, RRID:SCR_001876) [27], and SAMtools v1.4 [26]. We implemented HaplotypeCaller function of GATK v4.0 [27] for SNP detection and obtained an SNP data-set of the 150 individuals by CombineGVCFs, GenotypeGVCFs and SelectVariants module. For the original SNP data-set, we used VCFtools v0.1.13 (VCFtools, RRID:SCR_001235) [28] for quality control with the following standards: (i) 3X < mean sequencing depth (over all included individuals); (ii) allele frequency more than 0.05; (iii) maximum missing rate < 0.1; and (iv) only two alleles. Finally, we detected 36,488,048 informative SNPs.

We combined the re-sequencing data from above 150 pigs and the remaining re-sequencing data from 154 Eurasian pigs from the pig FarmGTEx project. We used PLINK v1.9 [29] to perform quality control on all 304 individuals, applying the following criteria: (i) allele frequency between 0.05; (ii) maximum missing rate < 0.1. Finally, a total of 24,523,019 informative SNPs covering 304 Eurasian pig breeds were implemented for subsequent analysis.

### DLW pigs specific population structure identification

To investigate population stratification among DLW pigs, French Large White pigs (FLW) and Netherlandish Large White pigs (NLW), we integrated a SNP data-set covering all above three populations. We downloaded the BAM files of NLW pigs from the NCBI SRA database (SRA number: ERP001813), a total of 12 individuals whose individual numbers are shown in Supplementary Table S9. We used the standard SNP calling protocol mentioned above to get informative SNPs. The data for 39 FLW pigs were genotyped with 50 K chip genotyping, and 44 DLW pigs were sequenced in this study. By identifying common SNPs among the three populations, we obtained a total of 19,373 informative SNPs for 95 pigs. We then utilized the “--pca” command from PLINK v1.9 [29] to calculate the principal components, selecting the first two for plotting.

To analyze the differences in genetic structure between Eurasian pig breeds, including TL pigs and DLW pigs, we utilized a re-sequencing dataset consisting of 24,523,019 informative SNPs from 304 Eurasian pig breeds. Firstly, we converted the PLINK format file into a MEGA format file and applied the p-distance algorithm under the neighbor-joining tree module to construct a neighbor-joining phylogenetic (NJ) tree by MEGA v6 (MEGA, RRID:SCR_000667) [30]. The same algorithm was applied to construct the NJ tree for the important candidate introgressed region located in SSC16: 33,160 kb − 33,275 kb region. Then, we used the command “--pca” from PLINK v1.9 [29] to calculate the principal components of the 304 pigs. The first two principal components were selected.

### Pairwise IBD detection

To identify the genomic regions that introgressed from TL into DLW pigs, we performed relative identity-by-descent (rIBD) analysis. We extracted re-sequencing data from 203 pigs (including only TL, DLW, and EUW populations) out of a total of 304 pigs. This subset comprised 50 Erhualian pigs, 56 Meishan pigs, 34 Jiaxing Black pigs, 44 DLW pigs, and 19 EUW pigs. We then re-ran the quality control using PLINK v1.9 [29] with the code “--maf 0.05 --geno 0.1”. After quality control, a total of 203 pigs with 19,720,401 informative SNPs were identified. The frequencies of shared haplotypes between DLW and TL pigs, or between DLW and EUW pigs was estimated by per 10,000-bp bins using IBDLD v3.37 [31], respectively. The parameters were set as: “-plinkbf_int file_name -method GIBDLD -ploci 10 -nthreads 38 -step 0 -hiddenstates 3 -segment --length 150”. The normalized IBD (nIBD) for a single pig group is calculated as: nIBD = cIBD/tIBD (where cIBD = count of all haplotypes IBD between DLW and one pig group and tIBD = total pairwise comparisons between DLW and one pig group). rIBD between two pig groups is calculated as: rIBD = nIBD_TL_–nIBD_EUW_.

The rIBD values range from − 1 to 1. Higher values indicate that DLW pigs share more homologous haplotypes with TL pigs, while lower values indicate that they share more homologous haplotypes with EUW pigs. We set the threshold at the top 0.5% rIBD value (rIBD = 0.052) to identify the rIBD regions for TL pigs introgressing into DLW pigs. We merged rIBD regions that were adjacent to each other with less than 200 kb, identifying a total of 37 introgressed regions (Supplementary Table S1).

For the identification of genomic regions introgressed from SCD into DLW pigs, we extracted re-sequencing data from 29 SCD pigs, 44 DLW pigs and 19 EUW pigs, and obtained 18,632,642 informative SNPs from 92 pigs according to the same quality control principles. The rIBD formula was used: rIBD = nIBD_SCD_-nIBD_EUW_. The threshold for identifying the rIBD regions for SCD pigs introgressing into DLW pigs was set at the top 0.5% rIBD value (rIBD = 0.091). We were able to identify a total of 42 introgressed regions (Supplementary Table S2).

### Annotation protein-coding genes and KEGG-enrichment analysis

The identification of protein-coding genes in rIBD regions and related upstream and downstream 200 kb region was conducted based on the Sus_scrofa assembly 11.1. The KEGG pathways (www.kegg.jp/kegg/kegg1.html) [32] in which these genes are enriched in were determined using the KOBAS (http://kobas.cbi.pku.edu.cn/kobas3) [33] based on corresponding human orthologous Ensembl IDs. Multiple tests were performed for KEGG terms using the FDR method. Pathways with an FDR < 0.05 were considered significantly enriched.

### Selective sweep analysis

To identify specific genomic regions which are different between TL and other Asian domestic and wild pigs, we implemented 106 TL pigs as group 1, all 72 pigs from Southern and Southwestern China as group 2, and 29 Asian wild boars as group 3 for locus-specific branch length (LSBL) analysis. The LSBL statistics were computed as previously reported [34]. For Fst analysis to determine the differential genomic regions between DLW and other pig breeds, we compared 44 DLW pigs with 106 TL pigs, 19 EUW pigs and 29 SCD pigs, respectively. Additionally, we examined the polymorphism of DLW and EUW pigs’ genome respectively by using Z-transformed heterozygosity (Zhet) statistics. Heterozygosity (Het) was calculated by the formula: Het = 2p*(1 - p), where p is the reference allele frequency in a particular population. We then standardized the Het values into Z-scores (Zhet) by adjusting each value with the overall mean and standard deviation. All of the aforementioned analyses for selection signal were conducted using a 10 kb window and a Z-test with a significance threshold of *P* < 0.05 was employed. We then showcased the statistic mentioned above in the SC16: 33,160 kb − 33,275 kb region in order to identify the differences in allele frequencies and genomic polymorphisms among various pig breeds in this 115 kb candidate introgressed region.

### Haplotype analysis

We cited Chen et al. [10] to confirm the association between haplotypes in candidate regions under selection and the estimated breeding value of total number of litter size (TNB_EBV). For the SSC16: 33,160 kb − 33,275 kb region (666 SNPs) among diversity breeds, we phased haplotypes using the fastPhase function in Beagle v4.0 (BEAGLE, RRID:SCR_001789) and to detect IBD regions in each individual using the fastIBD function [35]. We then used R package “pheatmap” for haplotype heat-map construction. We also used R package “pegas” to construct haplotype network map using filtered SNPs (104 SNPs, the absolute frequency difference between the population of DLW and EUW greater than 0.8).

We randomly selected 2 tag SNP loci that can distinguish the introgressed haplotypes (hap_1, hap_3, hap_5 and hap_9) from the other haplotype in the SSC16: 33,160 kb − 33,275 kb region to construct haplotypes (Fig. 3I). The TL pigs major haplotype is defined as introgressed Q haplotype and the others are unintrogressed q haplotype. We applied HIBLUP software [36] to calculate TNB_EBV using the following model:

$${\bf{y}}\,{\bf{ = }}\,{\bf{X\beta }}\,{\bf{ + }}\,{\bf{Zu}}\,{\bf{ + }}\,{\bf{e}}$$

where **y** is a vector of litter size (total number of born), **β** is a vector of fixed effects, including parity, year-season, and **X** is an design matrix of fixed effects. **u** is a vector of random effects, including random effects of mating boars, permanent environmental effects and additive genetic effect of sows. Additive genetic effect assumed to be normally distributed N(0, **A**σ_u_^2^), where **A** matrix is pedigree-based numerator relationship matrix, and σ_u_^2^ is the additive genetic variance. Mating boars random effects assumed to be normally distributed N(0, **A**σ_m_^2^), where **A** matrix is pedigree-based numerator relationship matrix, and σ_m_^2^ is the mating boars random effects variance. Permanent environmental random effects assumed to be normally distributed N(0, **I**σ_p_^2^), where **I** is the identity matrix and σ_p_^2^ is the permanent environmental random effects variance. **Z** is design matrix for the random effects. And **e** is a vector of random residuals that is normally distributed N(0, **I**σ_e_^2^), where **I** is the identity matrix and σ_e_^2^ is the residual variance.

Finally, we used a one-way ANOVA to test the association between haplotypes and TNB_EBV in Meishan pigs, Erhualian pigs and two DLW populations, respectively.

### Transcriptome sequencing and analysis

According to the estimated breeding value, we chose four high farrowing Erhualian pigs and four low farrowing Erhualian pigs on day 96 for RNA-seq analysis of the endometrium and myometrium. Additionally, based on the estimated breeding value, we selected another four high farrowing Erhualian pigs and four low farrowing Erhualian pigs on day 182 for RNA-seq analysis of the endometrium, myometrium, and ovary. The extraction of total RNA from the tissues were performed by the traditional trziol method. For each sample, one µg of total RNA was used for the construction of a 2 × 150 bp paired-end mRNA sequencing library with the NEBNext® UltraTM RNA Library Prep kit for Illumina® (NEB, USA). RNA sequencing reactions were conducted on the Illumina Hiseq-PE150 platform. The average output was 6 Gb per library. We used FastQC software [24] for quality control. STAR v2.7.1 (STAR, RRID:SCR_004463) [37] and featureCounts v2.0.0 (featureCounts, RRID:SCR_012919) [38] were used to align the clean reads to Sus_scrofa_11.1 reference genome. We subsequently transformed gene expression into FPKM values and conducted t-tests to ascertain if there was differential expression of the three genes *NDUFS4*, *ARL15*, and *ENSSSCG00000041656* in various tissues between the high and low Erhualian farrowing groups.

### Electronic supplementary material

Below is the link to the electronic supplementary material.

**Supplementary Material 1**: **Supplementary Fig S1**: Venn diagram of the introgressed genes from Asian pigs between DLW pigs and FLW pigs or NLW pigs. **Supplementary Fig S2**: Venn diagram of the introgressed genes from TL and SCD pigs into DLW pigs. **Supplementary Table S1**: List of introgressed regions from TL into DLW pigs. **Supplementary Table S2**: List of introgressed regions from SCD into DLW pigs. **Supplementary Table S3**: KEGG functional enrichment of protein_coding genes annotated by introgressed regions from TL into DLW pigs. **Supplementary Table S4**: Most significant introgressed regions annotated gene from TL into DLW pigs. **Supplementary Table S5**: Haplotype statistics. **Supplementary Table S6**: Association analysis between introgressed haplotypes and TNB_EBV. **Supplementary Table S7**: Statistics of sequencing of samples. **Supplementary Table S8**: List of 304 pig individuals information. **Supplementary Table S9**: List of downloaded samples

## Data Availability

The genotype datasets generated and/or analyzed during the current study are available in the Figshare repository, 10.6084/m9.figshare.23940450.v1. The RNA-seq datasets generated and/or analyzed during the current study are available from the corresponding author upon reasonable request.

## List of abbreviations

TL: Taihu Lake region
FLW: French Large White
DLW: Danish Large White
SCD: Southern China indigenous pigs
SWCD: Southwestern China indigenous pigs
EUW: European wild boar
ASW: Asian wild boar
NLW: Netherlandish Large White
NJ: Neighbor-joining phylogenetic tree
rIBD: Relative identity-by-descent
nIBD: Normalized IBD
cIBD: Count of all haplotypes IBD
tIBD: Total pairwise comparisons IBD
Zhet: Z-transformed heterozygosity
Het: Heterozygosity
TNB_EBV: Estimated breeding value of total number of litter size
PCA: Principal component analysis
LSBL: Locus-specific branch length
Fst: Genetic differentiation coefficients
