# Supplementary material for: Introgression of pigs in Taihu Lake region possibly contributed to the improvement of fertility in Danish Large White pigs
Source: BMC Genomics. 2023 Dec 4;24:733. doi: 10.1186/s12864-023-09860-x (PMC10694980; doi:10.1186/s12864-023-09860-x)
Supplement: Supplementary file 1 — Supplementary Material 1: Supplementary Fig S1: Venn diagram of the introgressed genes from Asian pigs between DLW pigs and FLW pigs or NLW pigs. Supplementary Fig S2: Venn diagram of the introgressed genes from TL and SCD pigs into DLW pigs. Supplementary Table S1: List of introgressed regions from TL into DLW pigs. Supplementary Table S2: List of introgressed regions from SCD into DLW pigs. Supplementary Table S3: KEGG functional enrichment of protein_coding genes annotated by introgressed regions from TL into DLW pigs. Supplementary Table S4: Most significant introgressed regions annotated gene from TL into DLW pigs. Supplementary Table S5: Haplotype statistics. Supplementary Table S6: Association analysis between introgressed haplotypes and TNB_EBV. Supplementary Table S7: Statistics of sequencing of samples. Supplementary Table S8: List of 304 pig individuals information. Supplementary Table S9: List of downloaded samples [file 12864_2023_9860_MOESM1_ESM.docx]

**Overview List of Supplementary Figures**

**Supplementary Fig S1.** Venn diagram of the introgressed genes from Asian pigs between DLW pigs and FLW pigs or NLW pigs.

**Supplementary Fig S2.** Venn diagram of the introgressed genes from TL and SCD pigs into DLW pigs.

**Overview List of Supplementary Tables**

**Supplementary Table S1.** List of introgressed regions from TL into DLW pigs.

**Supplementary Table S2.** List of introgressed regions from SCD into DLW pigs.

**Supplementary Table S3.** KEGG functional enrichment of protein_coding genes annotated by introgressed regions from TL into DLW pigs.

**Supplementary Table S4.** Most significant introgressed regions annotated gene from TL into DLW pigs.

**Supplementary Table S5.** Haplotype statistics.

**Supplementary Table S6.** Association analysis between introgressed haplotypes and TNB_EBV.

**Supplementary Table S7.** Statistics of sequencing of samples.

**Supplementary Table S8.** List of 304 pig individuals information

**Supplementary Table S9.** List of downloaded samples.


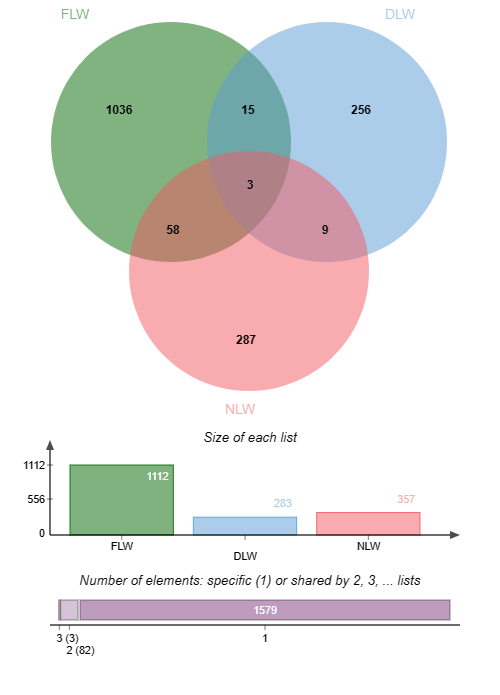


**Figure S1.** Venn diagram of the introgressed genes from Asian pigs between DLW pigs and FLW pigs or NLW pigs.


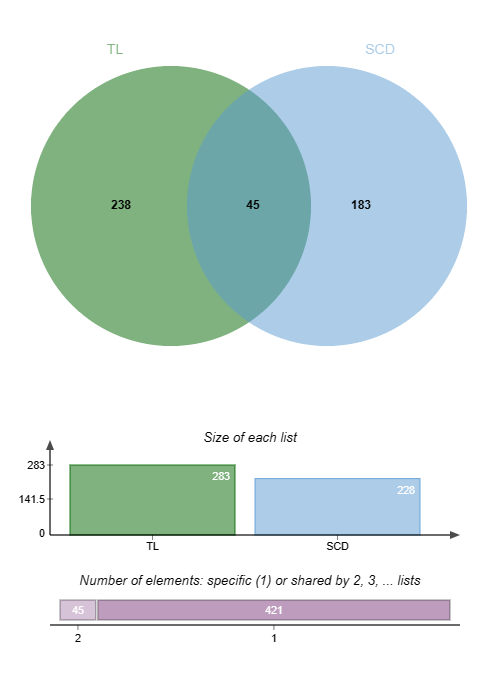


**Figure S2.** Venn diagram of the introgressed genes from TL and SCD pigs into DLW pigs.

**Table S1.** List of introgressed regions from TL into DLW pigs

| **CHR** | **POS-BEGAN/ bp** | **POS-END/bp** | **Average rIBD value** | **rIBD peak value** |
| --- | --- | --- | --- | --- |
| 1 | 47520001 | 48330000 | 0.203 | 0.411 |
| 1 | 49460001 | 49690000 | 0.067 | 0.086 |
| 1 | 55960001 | 56120000 | 0.111 | 0.119 |
| 1 | 60770001 | 60820000 | 0.077 | 0.085 |
| 1 | 64370001 | 65560000 | 0.168 | 0.313 |
| 1 | 68060001 | 68390000 | 0.174 | 0.218 |
| 1 | 68710001 | 69170000 | 0.085 | 0.101 |
| 1 | 69380001 | 69820000 | 0.134 | 0.198 |
| 1 | 72650001 | 72860000 | 0.053 | 0.053 |
| 1 | 81950001 | 82120000 | 0.078 | 0.082 |
| 1 | 82330001 | 82670000 | 0.072 | 0.100 |
| 1 | 89200001 | 89360000 | 0.082 | 0.105 |
| 1 | 264280001 | 265610000 | 0.073 | 0.122 |
| 1 | 266050001 | 266090000 | 0.071 | 0.073 |
| 2 | 59050001 | 59160000 | 0.055 | 0.055 |
| 2 | 66070001 | 66100000 | 0.102 | 0.131 |
| 2 | 72980001 | 73430000 | 0.087 | 0.128 |
| 2 | 77310001 | 77620000 | 0.095 | 0.186 |
| 2 | 78280001 | 78930000 | 0.073 | 0.117 |
| 2 | 80510001 | 80550000 | 0.055 | 0.056 |
| 3 | 115650001 | 116900000 | 0.100 | 0.122 |
| 6 | 25000001 | 25070000 | 0.056 | 0.057 |
| 8 | 96760001 | 96960000 | 0.063 | 0.068 |
| 9 | 98570001 | 99480000 | 0.116 | 0.189 |
| 9 | 99710001 | 100360000 | 0.108 | 0.145 |
| 13 | 155200001 | 155380000 | 0.157 | 0.189 |
| 14 | 49200001 | 49270000 | 0.055 | 0.057 |
| 15 | 12180001 | 12350000 | 0.066 | 0.070 |
| 15 | 26060001 | 26290000 | 0.072 | 0.076 |
| 15 | 59030001 | 59140000 | 0.064 | 0.069 |
| 15 | 59460001 | 59690000 | 0.068 | 0.073 |
| 15 | 61200001 | 61240000 | 0.061 | 0.062 |
| 15 | 102620001 | 102820000 | 0.057 | 0.073 |
| 15 | 104640001 | 104970000 | 0.075 | 0.090 |
| 15 | 105240001 | 105450000 | 0.058 | 0.058 |
| 15 | 112380001 | 112410000 | 0.055 | 0.055 |
| 16 | 32880001 | 33330000 | 0.140 | 0.208 |

**Table S2.** List of introgressed regions from SCD into DLW pigs

| **CHR** | **POS-BEGAN/ bp** | **POS-END/bp** | **Average rIBD value** | **rIBD peak value** |
| --- | --- | --- | --- | --- |
| 1 | 510001 | 520000 | 0.165 | 0.220 |
| 1 | 38280001 | 38420000 | 0.100 | 0.104 |
| 1 | 57790001 | 57980000 | 0.115 | 0.126 |
| 1 | 60800001 | 60830000 | 0.103 | 0.121 |
| 1 | 81560001 | 82040000 | 0.111 | 0.134 |
| 1 | 82330001 | 84140000 | 0.142 | 0.236 |
| 1 | 88490001 | 89070000 | 0.127 | 0.167 |
| 1 | 90140001 | 90320000 | 0.160 | 0.187 |
| 2 | 91180001 | 91530000 | 0.239 | 0.347 |
| 2 | 109720001 | 109910000 | 0.095 | 0.133 |
| 3 | 31790001 | 31950000 | 0.123 | 0.141 |
| 5 | 33660001 | 33880000 | 0.150 | 0.158 |
| 8 | 64300001 | 64940000 | 0.116 | 0.129 |
| 8 | 101640001 | 101940000 | 0.120 | 0.134 |
| 8 | 108300001 | 108480000 | 0.106 | 0.111 |
| 8 | 136990001 | 137140000 | 0.105 | 0.107 |
| 8 | 137580001 | 138610000 | 0.194 | 0.426 |
| 9 | 85040001 | 85090000 | 0.091 | 0.092 |
| 12 | 28690001 | 28780000 | 0.104 | 0.107 |
| 13 | 20180001 | 20540000 | 0.228 | 0.309 |
| 13 | 33160001 | 33310000 | 0.097 | 0.105 |
| 13 | 69510001 | 69590000 | 0.093 | 0.093 |
| 13 | 84200001 | 84260000 | 0.104 | 0.114 |
| 13 | 105870001 | 105960000 | 0.093 | 0.095 |
| 13 | 155130001 | 155300000 | 0.126 | 0.130 |
| 13 | 157550001 | 157730000 | 0.100 | 0.109 |
| 13 | 160900001 | 161010000 | 0.113 | 0.122 |
| 13 | 173160001 | 173320000 | 0.106 | 0.123 |
| 14 | 48450001 | 48540000 | 0.098 | 0.102 |
| 14 | 49270001 | 49520000 | 0.098 | 0.107 |
| 14 | 101680001 | 101870000 | 0.099 | 0.115 |
| 15 | 85880001 | 86620000 | 0.115 | 0.131 |
| 15 | 91800001 | 92040000 | 0.104 | 0.147 |
| 15 | 95080001 | 95580000 | 0.102 | 0.110 |
| 15 | 96010001 | 96190000 | 0.102 | 0.103 |
| 15 | 96650001 | 96960000 | 0.103 | 0.129 |
| 15 | 98210001 | 99240000 | 0.097 | 0.112 |
| 15 | 99510001 | 99780000 | 0.098 | 0.128 |
| 15 | 100740001 | 103410000 | 0.123 | 0.137 |
| 15 | 104770001 | 105300000 | 0.102 | 0.109 |
| 16 | 19030001 | 19230000 | 0.123 | 0.137 |
| 16 | 29840001 | 29960000 | 0.140 | 0.173 |

**Table S3.** KEGG functional enrichment of protein_coding genes annotated by introgressed regions from TL into DLW pigs

| **KEGG Term** | **Input number** | **Background number** | **Corrected P-Value** | | **Input Genes** |
| --- | --- | --- | --- | --- | --- |
| Protein processing in endoplasmic reticulum | 9 | 165 | 0.0005 | UBE2J1\|HSPA5\|RAD23A\|CALR\|HSPA4L\|LMAN2\|MAPK9\|CANX\|UGGT1 | |
| GABAergic synapse | 6 | 89 | 0.0031 | TRAK2\|GABRR1\|GABRR2\|CACNA1A\|GLS\|GNAI1 | |
| Parkinson disease | 7 | 142 | 0.0043 | ADORA2A\|UBE2J1\|ATP5F1D\|NDUFS4\|NDUFA11\|SLC25A31\|GNAI1 | |
| Retrograde endocannabinoid signaling | 7 | 148 | 0.0050 | GABRR1\|GABRR2\|NDUFS4\|NDUFA11\|CACNA1A\|MAPK9\|GNAI1 | |
| Necroptosis | 7 | 162 | 0.0073 | SQSTM1\|STAT4\|STAT1\|CASP8\|CFLAR\|SLC25A31\|MAPK9 | |
| Glutamatergic synapse | 6 | 114 | 0.0075 | CACNA1A\|GRIK2\|GRIN3B\|HOMER3\|GLS\|GNAI1 | |
| Nicotine addiction | 4 | 40 | 0.0086 | GABRR1\|GABRR2\|GRIN3B\|CACNA1A | |
| Huntington disease | 7 | 193 | 0.0136 | ATP5F1D\|CASP8\|POLR2E\|POLR2D\|NDUFS4\|NDUFA11\|SLC25A31 | |
| Morphine addiction | 5 | 91 | 0.0149 | GRK6\|GABRR1\|GABRR2\|CACNA1A\|GNAI1 | |
| Rap1 signaling pathway | 7 | 210 | 0.0194 | ADORA2A\|RGS14\|EFNA2\|PFN3\|FLT4\|FGF22\|GNAI1 | |
| Chagas disease (American trypanosomiasis) | 5 | 103 | 0.0225 | CFLAR\|CALR\|MAPK9\|CASP8\|GNAI1 | |
| Hepatitis B | 6 | 163 | 0.0247 | STAT4\|STAT1\|CASP8\|CASP10\|HGF\|MAPK9 | |
| Shigellosis | 4 | 68 | 0.0302 | MAPK9\|PFN3\|ARPC5L\|ATG5 | |
| RIG-I-like receptor signaling pathway | 4 | 70 | 0.0318 | CASP10\|MAPK9\|CASP8\|ATG5 | |
| Metabolic pathways | 20 | 1433 | 0.0387 | QRSL1\|GAMT\|ISYNA1\|GALNT13\|GCDH\|TRAK2\|CFD\|ATP5F1D\|LTC4S\|ACER1\|ME1\|GPX4\|ACSBG2\|MGAT4B\|GLS\|NDUFS4\|GFPT2\|DSE\|B4GALT7\|NDUFA11 | |
| FoxO signaling pathway | 5 | 132 | 0.0411 | STK11\|HGF\|HOMER3\|MAPK9\|PLK4 | |

**Table S4.** Most significant introgressed regions annotated gene from TL into

DLW pigs

| **CHR** | **POS-BEGAN/bp** | **POS-END/bp** | **Average rIBD value** | **Annotated protein-coding gene (200 kb upstream and downstream)** |
| --- | --- | --- | --- | --- |
| 1 | 47550001 | 48190000 | 0.273606925 |  |
| 1 | 64380001 | 64560000 | 0.277368611 | KLHL32\|MMS22L |
| 1 | 64680001 | 64840000 | 0.142972488 |  |
| 1 | 68060001 | 68390000 | 0.17924926 | GRIK2 |
| 1 | 69470001 | 69810000 | 0.14952636 |  |
| 2 | 73260001 | 73330000 | 0.128246753 | RFX2\|RANBP3\|CAPS\|VMAC\|NDUFA11\|NRTN\|DUS3L\|CATSPERD\|LONP1\|RPL36\|HSD11B1L\|MICOS13\|SAFB\|SAFB2\|TINCR\|ZNRF4 |
| 2 | 77590001 | 77620000 | 0.163493962 | POLR2E\|ARHGAP45\|ABCA7\|CNN2\|TMEM259\|GRIN3B\|WDR18\|ARID3A\|KISS1R\|R3HDM4\|MED16\|ELANE\|CFD\|PRTN3\|AZU1\|PLPPR3\|PTBP1\|MISP\|PALM\|PRSS57\|FSTL3\|RNF126\|FGF22\|POLRMT\|HCN2\|GZMM\|CDC34\|TPGS1\|MADCAM1\|ODF3L2 |
| 9 | 98700001 | 99340000 | 0.136378957 | HGF\|SEMA3C\|GNAT3 |
| 9 | 100010001 | 100320000 | 0.133079735 | GNAI1 |
| 13 | 155230001 | 155380000 | 0.165051834 |  |
| 16 | 33170001 | 33330000 | 0.205519481 | NDUFS4\|ARL15\|ENSSSCG00000041656 |

**Table S5.** Haplotype statistics

| **Haplotype** | **Breed** | | | | | |
| --- | --- | --- | --- | --- | --- | --- |
|  | **ASW/n** | **SWCD/n** | **SCD/n** | **TL/n** | **DLW/n** | **EUW/n** |
| Hap_1 | 0 | 0 | 0 | 124 | 74 | 0 |
| Hap_2 | 0 | 0 | 1 | 5 | 0 | 0 |
| Hap_3 | 0 | 0 | 0 | 17 | 0 | 0 |
| Hap_4 | 0 | 0 | 0 | 6 | 0 | 0 |
| Hap_5 | 0 | 0 | 0 | 2 | 0 | 0 |
| Hap_6 | 0 | 0 | 0 | 2 | 0 | 0 |
| Hap_7 | 0 | 0 | 9 | 1 | 0 | 0 |
| Hap_8 | 0 | 0 | 1 | 1 | 0 | 0 |
| Hap_9 | 0 | 0 | 0 | 2 | 0 | 0 |
| Hap_10 | 1 | 41 | 38 | 0 | 0 | 38 |
| Hap_11 | 0 | 3 | 1 | 0 | 0 | 0 |
| Hap_12 | 0 | 1 | 1 | 0 | 0 | 0 |
| Hap_13 | 5 | 0 | 0 | 0 | 0 | 0 |
| Hap_14 | 2 | 0 | 0 | 0 | 0 | 0 |
| Hap_15 | 0 | 4 | 0 | 0 | 0 | 0 |
| Hap_16 | 0 | 2 | 0 | 0 | 0 | 0 |
| Hap_17 | 0 | 8 | 0 | 0 | 0 | 0 |
| Hap_18 | 0 | 3 | 0 | 0 | 0 | 0 |
| Hap_19 | 0 | 2 | 0 | 0 | 0 | 0 |
| Hap_20 | 0 | 2 | 0 | 0 | 0 | 0 |
| Hap_21 | 12 | 0 | 0 | 0 | 0 | 0 |
| Hap_22 | 2 | 0 | 0 | 0 | 0 | 0 |

**Table S6.** Association analysis between introgressed haplotypes and TNB_EBV

| **Population** | **Haplotype** | **TNB_EBV** | **Sample/n** | ***P***-value |
| --- | --- | --- | --- | --- |
| MS | qq | -0.1768±0.3784 | 76 | <0.001 |
|  | Qq | -0.0197±0.2886 | 80 |  |
|  | QQ | 0.1201±0.3020 | 125 |  |
| EHL | qq | -0.1004±0.3081 | 35 | 0.035 |
|  | Qq | -0.0423±0.4081 | 104 |  |
|  | QQ | 0.0761±0.4559 | 112 |  |
| DLW-1 | Qq | -0.3432±0.4928 | 13 | 0.028 |
|  | QQ | 0.0479±0.6234 | 196 |  |
| DLW-2 | Qq | -0.2628±1.0597 | 57 | 0.011 |
|  | QQ | 0.1279±1.0450 | 276 |  |

**Table S7.** Statistics of sequencing of samples

| **Sample ID** | **Population** | **Sequencing Depth** | **Sequencing genome coverage** |
| --- | --- | --- | --- |
| 1009 | MS | 9.25 | 98.79 |
| M1659 | MS | 9.54 | 98.46 |
| 1897 | MS | 10.26 | 98.68 |
| M1516 | MS | 10.56 | 98.61 |
| 388 | MS | 9.73 | 98.55 |
| CM2 | MS | 10.75 | 98.57 |
| 545 | MS | 9.50 | 98.54 |
| M3344 | MS | 9.72 | 98.69 |
| M3740 | MS | 10.41 | 98.66 |
| 1225 | MS | 10.93 | 98.63 |
| M3490 | MS | 9.32 | 98.57 |
| 2906 | MS | 10.16 | 98.77 |
| M1003 | MS | 9.47 | 98.65 |
| 1488 | MS | 10.98 | 98.65 |
| M1083 | MS | 9.67 | 98.50 |
| 6683 | MS | 10.52 | 98.63 |
| CM3124 | MS | 9.55 | 98.46 |
| CM1576 | MS | 10.78 | 98.76 |
| 6663 | MS | 10.52 | 98.55 |
| 1144 | MS | 11.82 | 97.84 |
| 1238 | MS | 11.86 | 97.95 |
| 1316 | MS | 11.39 | 96.08 |
| 1426 | MS | 10.20 | 96.51 |
| 1546 | MS | 11.35 | 98.33 |
| 3240 | MS | 11.98 | 98.07 |
| CM116 | MS | 11.71 | 98.21 |
| CM2588 | MS | 10.54 | 97.07 |
| CM3126 | MS | 11.83 | 98.22 |
| SM1088 | MS | 10.62 | 98.26 |
| 2860 | MS | 10.25 | 98.65 |
| 4083 | MS | 10.01 | 98.62 |
| 5234 | MS | 10.70 | 98.7 |
| 7733 | MS | 9.07 | 98.69 |
| 1243 | MS | 9.28 | 98.66 |
| 753 | MS | 9.31 | 98.72 |
| 3213 | MS | 9.49 | 98.69 |
| 2957 | MS | 9.72 | 98.73 |
| 6747 | MS | 9.82 | 98.64 |
| KM2604 | MS | 10.52 | 98.69 |
| 2834 | MS | 9.63 | 98.82 |
| 8828 | MS | 9.20 | 98.67 |
| 2956 | MS | 9.49 | 98.73 |
| 3582 | MS | 10.77 | 98.84 |
| KM2688 | MS | 10.46 | 98.71 |
| 1139 | MS | 10.31 | 98.62 |
| 402 | MS | 10.74 | 98.11 |
| 4478 | MS | 10.93 | 98.22 |
| 5622 | MS | 11.41 | 98.35 |
| 7558 | MS | 12.02 | 98.19 |
| 7902 | MS | 12.06 | 98.40 |
| 8144 | MS | 10.39 | 98.21 |
| 8554 | MS | 12.45 | 98.38 |
| 8578 | MS | 11.25 | 97.71 |
| 8910 | MS | 12.03 | 98.02 |
| 9550 | MS | 10.68 | 98.25 |
| KM180 | MS | 10.28 | 96.2 |
| 10 | EHL | 11.92 | 98.17 |
| 1713 | EHL | 10.82 | 97.89 |
| 1870 | EHL | 11.95 | 98.56 |
| 2016 | EHL | 11.19 | 98.45 |
| 2090 | EHL | 10.29 | 96.34 |
| 2148 | EHL | 11.42 | 97.45 |
| 22 | EHL | 10.54 | 98.48 |
| 2216 | EHL | 9.98 | 98.33 |
| 2498 | EHL | 10.64 | 98.67 |
| 2550 | EHL | 11.12 | 97.23 |
| 2568 | EHL | 10.54 | 97.48 |
| 3241 | EHL | 10.28 | 98.13 |
| 345-1 | EHL | 9.73 | 96.65 |
| 3604 | EHL | 10.80 | 96.99 |
| 4256 | EHL | 9.85 | 97.45 |
| 4267 | EHL | 11.23 | 98.38 |
| 4613 | EHL | 10.87 | 97.66 |
| 5003 | EHL | 10.64 | 97.45 |
| 5436 | EHL | 10.55 | 97.45 |
| 5813 | EHL | 11.23 | 98.58 |
| 6452 | EHL | 9.69 | 98.74 |
| 6474 | EHL | 11.90 | 98.44 |
| 6733 | EHL | 9.68 | 98.29 |
| 6741 | EHL | 10.58 | 97.89 |
| 6755 | EHL | 10.94 | 98.13 |
| 7572 | EHL | 10.89 | 96.59 |
| 7954 | EHL | 10.65 | 96.77 |
| 8558 | EHL | 10.78 | 97.57 |
| 8582 | EHL | 11.18 | 98.46 |
| 890 | EHL | 12.21 | 97.55 |
| CZE1760 | EHL | 11.56 | 97.94 |
| CZE1888 | EHL | 10.89 | 96.92 |
| CZE467 | EHL | 11.56 | 98.19 |
| CZE5800 | EHL | 9.88 | 98.46 |
| CZE8212 | EHL | 10.98 | 98.42 |
| E1210 | EHL | 10.10 | 97.83 |
| E2309 | EHL | 10.56 | 96.67 |
| E2501 | EHL | 10.32 | 96.54 |
| E2582 | EHL | 10.34 | 97.99 |
| E3262 | EHL | 10.56 | 98.36 |
| E3353 | EHL | 11.34 | 98.57 |
| E3377 | EHL | 9.76 | 97.64 |
| E3491 | EHL | 9.84 | 97.59 |
| E3940 | EHL | 10.65 | 98.51 |
| E4396 | EHL | 10.41 | 96.67 |
| E6527 | EHL | 9.88 | 98.44 |
| E7646 | EHL | 10.01 | 98.28 |
| E8582 | EHL | 10.56 | 97.59 |
| E9370 | EHL | 10.06 | 97.45 |
| STE3576 | EHL | 10.41 | 97.89 |
| 291504-259 | DLW | 11.74 | 96.98 |
| 302105-257 | DLW | 13.62 | 98.43 |
| 321602-254 | DLW | 11.53 | 98.61 |
| 321605-270 | DLW | 12.74 | 98.18 |
| 340609-258 | DLW | 12.96 | 98.26 |
| 342211-279 | DLW | 14.00 | 98.45 |
| 366311-278 | DLW | 26.12 | 98.61 |
| 40467-282 | DLW | 12.31 | 98.72 |
| 40473-274 | DLW | 12.94 | 98.42 |
| 40624-246 | DLW | 13.21 | 97.65 |
| 40711-271 | DLW | 13.49 | 97.16 |
| 40734-269 | DLW | 12.36 | 98.59 |
| 40754-249 | DLW | 10.26 | 97.66 |
| 40818-277 | DLW | 13.16 | 97.08 |
| 40948-243 | DLW | 13.84 | 98.37 |
| 40977-245 | DLW | 15.20 | 98.08 |
| 41039-268 | DLW | 11.18 | 98.07 |
| 41040-275 | DLW | 13.21 | 98.71 |
| 41113-242 | DLW | 13.25 | 98.52 |
| 41121-267 | DLW | 11.77 | 97.05 |
| 41339-253 | DLW | 12.41 | 96.94 |
| 41564-250 | DLW | 11.15 | 96.92 |
| 41697-244 | DLW | 11.51 | 98.41 |
| 41830-247 | DLW | 11.18 | 98.17 |
| 41851-266 | DLW | 9.99 | 97.61 |
| 41852-261 | DLW | 11.10 | 96.93 |
| 41863-241 | DLW | 14.13 | 97.85 |
| 41897-263 | DLW | 12.08 | 98.15 |
| 5213-256 | DLW | 13.74 | 98.27 |
| 5378-272 | DLW | 13.89 | 97.16 |
| 5414-264 | DLW | 12.87 | 97.64 |
| 6385-260 | DLW | 11.43 | 98.04 |
| 6474-248 | DLW | 12.33 | 97.97 |
| 6539-265 | DLW | 12.02 | 97.63 |
| 6608-280 | DLW | 11.86 | 98.50 |
| 6623-255 | DLW | 10.18 | 98.42 |
| 6777-262 | DLW | 10.18 | 96.84 |
| 7249-276 | DLW | 14.23 | 97.15 |
| 7268-283 | DLW | 15.41 | 96.63 |
| 7753-252 | DLW | 10.35 | 98.24 |
| 8639-273 | DLW | 16.75 | 98.10 |
| 9638-284 | DLW | 12.27 | 97.93 |
| 9667-281 | DLW | 12.49 | 97.65 |
| 9848-251 | DLW | 11.77 | 97.59 |

**Table S8.** List of 304 pig individuals information

| category | Breed | Abbreviation | Sample size |
| --- | --- | --- | --- |
| Taihu Lake region | Erhualian | EHL | 50 |
|  | Meishan | MS | 56 |
|  | Jiaxing Black | JXB | 34 |
| Southern China indigenous pigs | Bamaxiang | BMX | 6 |
|  | Congjiangxiang | CJX | 16 |
|  | Neijiang | NJ | 9 |
|  | Wuzhishan | WZS | 6 |
| Southwestern China indigenous pigs | Diqing Tibetan pig | DQT | 8 |
|  | Gansu Tibetan pig | GST | 10 |
|  | Litang Tibetan pig | LTT | 3 |
|  | Linzhi Tibetan pig | LZT | 2 |
|  | Milin Tibetan pig | MLT | 4 |
|  | Rongchang | RC | 8 |
| Danish Large White | Danish Large White | DLW | 44 |
| European boar | European boar | EUW | 19 |
| Asian boar | Asian boar | ASW | 29 |

**Table S9.** List of downloaded samples

| **BioSample** | **Run** | **Experiment** | **Breed** |
| --- | --- | --- | --- |
| SAMEA1557415 | ERR173186 | ERX149149 | Netherlands Large White |
| SAMEA1557431 | ERR173187 | ERX149150 | Netherlands Large White |
| SAMEA1557402 | ERR173189 | ERX149152 | Netherlands Large White |
| SAMEA1557389 | ERR173190 | ERX149153 | Netherlands Large White |
| SAMEA1557435 | ERR173191 | ERX149154 | Netherlands Large White |
| SAMEA1557422 | ERR173192 | ERX149155 | Netherlands Large White |
| SAMEA1557406 | ERR173193 | ERX149156 | Netherlands Large White |
| SAMEA1557404 | ERR173194 | ERX149157 | Netherlands Large White |
| SAMEA1557383 | ERR173195 | ERX149158 | Netherlands Large White |
| SAMEA1557425 | ERR173196 | ERX149159 | Netherlands Large White |
| SAMEA1557427 | ERR173197 | ERX149160 | Netherlands Large White |
| SAMEA1557399 | ERR173198 | ERX149161 | Netherlands Large White |
